# Application of Continuous Local Antibiotic Perfusion for Postoperative Mediastinitis

**DOI:** 10.7759/cureus.98405

**Published:** 2025-12-03

**Authors:** Koki Yokawa, Taku Nakagawa, Makoto Kusakizako, Tomonori Higuma, Yosuke Tanaka, Kazunori Yoshida, Yoshihiro Oshima, Hidefumi Obo, Hidetaka Wakiyama

**Affiliations:** 1 Department of Cardiovascular Surgery, Kakogawa Central City Hospital, Kakogawa, JPN

**Keywords:** adult cardiac surgery, continuous local antibiotic perfusion, mediastinitis, postoperative complication, surgical site infection

## Abstract

Objectives: Postoperative mediastinitis after cardiovascular surgery remains a significant concern because it leads to prolonged hospitalization and increased mortality. Recently, continuous local antibiotic perfusion (CLAP) has been widely used as a new route of antimicrobial administration in orthopedic surgery. We herein report the outcomes of applying CLAP to mediastinitis after cardiovascular surgery.

Methods: Seven patients (mean age, 52±22 years) who underwent CLAP for mediastinitis after cardiovascular surgery at our hospital from May 2020 to December 2024 were enrolled and retrospectively analyzed. Pathogenic bacteria identified included methicillin-resistant Staphylococcus aureus in five patients and Pseudomonas in two patients. The wounds were closed with 18-24 Fr Salem sump tubes placed in the wound after appropriate debridement. A negative pressure continuous therapy device was connected to the main sump tube to maintain a negative pressure of 60 mmHg, with gentamicin at a concentration of 1.2 mg/mL perfused at a rate of 2 mL/h through the subroute.

Results: No hospital mortality occurred. All patients achieved sternal closure without severe adverse events. One patient underwent closure with an omentum flap in the early stages of CLAP implementation. The median length of CLAP was 14 (5-24) days. The mean duration of respiratory support and the duration until oral feeding were 2.5 and 3.3 days after CLAP initiation, respectively. No gentamicin-induced adverse events were observed. The three-year survival rate was 75%, whereas the reinfection-free rate was 100%.

Conclusion: CLAP can be easily introduced to facilitate early extubation, early ambulation, and early resumption of oral intake, which are beneficial for infection control.

## Introduction

Mediastinitis is a critical complication after cardiac and aortic surgeries via median sternotomy that can occasionally follow a highly severe clinical course [1]. Previous studies have demonstrated the effectiveness of continuous suction irrigation methods and the necessity of omental filling for treating mediastinitis [2,3]. However, continuous suction irrigation in an open-chest state or frequent open-chest washouts can significantly weaken the patient’s physical condition, leading to malnutrition, a decline in activities of daily living (ADLs), and, in some cases, death [4]. Treating infections requires not only systemic antibiotic administration but also a multidisciplinary approach that includes preventing nutritional decline and deterioration of ADLs. Moreover, although intravenous antibiotic administration has been the standard treatment for perioperative infections, whether such an approach is truly optimal and sufficient remains unclear. Recently, researchers in orthopedics have developed continuous local antibiotic perfusion (CLAP) as a treatment for contaminated wounds, such as open fractures, and have confirmed its effectiveness [5].

We therefore considered whether this method could be applied to mediastinitis following cardiac and great vessel surgeries. The present study reports our experience with the use of CLAP for postoperative mediastinitis. CLAP was intended to enable early sternal closure, facilitate prompt initiation of oral intake and rehabilitation, and serve as an effective treatment strategy for postoperative mediastinitis.

## Materials and methods

Patient selection and study design

Among the 685 patients who underwent cardiac and aortic surgeries via median sternotomy from May 2020 to November 2024, seven (1.0%) who were diagnosed with mediastinitis and treated with CLAP were included in this study. Mediastinitis was diagnosed based on postoperative fever, elevated inflammatory markers on blood tests, and imaging findings on computed tomography that suggested surgical site infection. The causative organism was identified through blood and wound cultures obtained during chest reopening. Additionally, 10 patients who received CLAP for suspected mediastinitis or postoperative wound infection were excluded after detecting no causative organisms on wound cultures.

This study was a retrospective, non-randomized observational study that included seven patients with postoperative mediastinitis treated with CLAP. The study period was set from May 2020 to November 2024 because the CLAP procedure was first introduced at our institution in 2020.

Continuous local antibiotic perfusion

**Figure 1:**
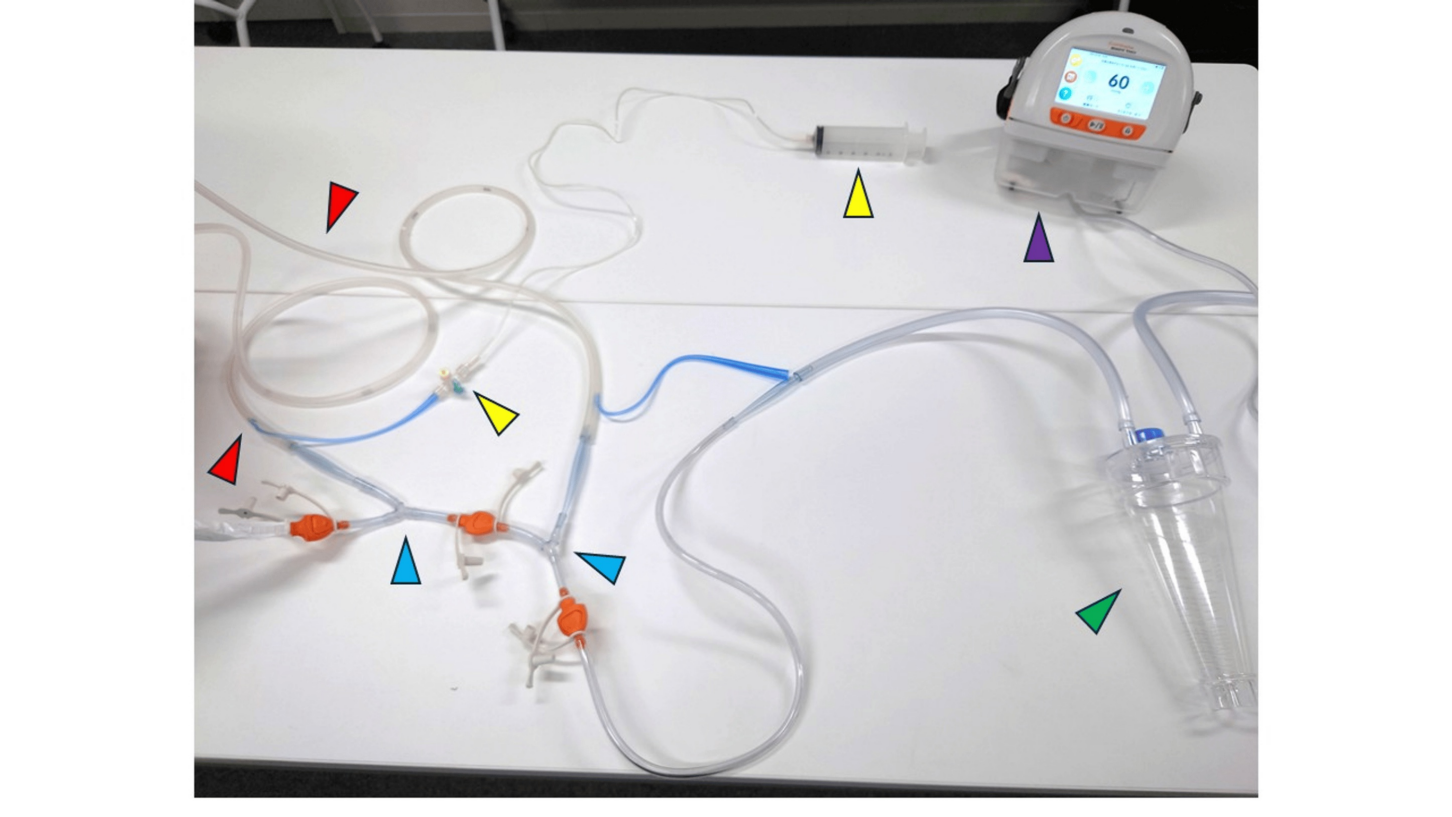
Schematic illustration of the CLAP system. Example of the connection setup for the CLAP system. The red arrowheads indicate the infusion lines for antibiotic administration, the yellow arrowheads show the three-way stopcocks for drug injection and flow adjustment, the blue arrowheads mark the Y-connectors, the green arrowhead indicates the vacuum drainage bottle, and the purple arrowhead shows the negative pressure control device. CLAP, continuous local antibiotic perfusion

**Figure 2:**
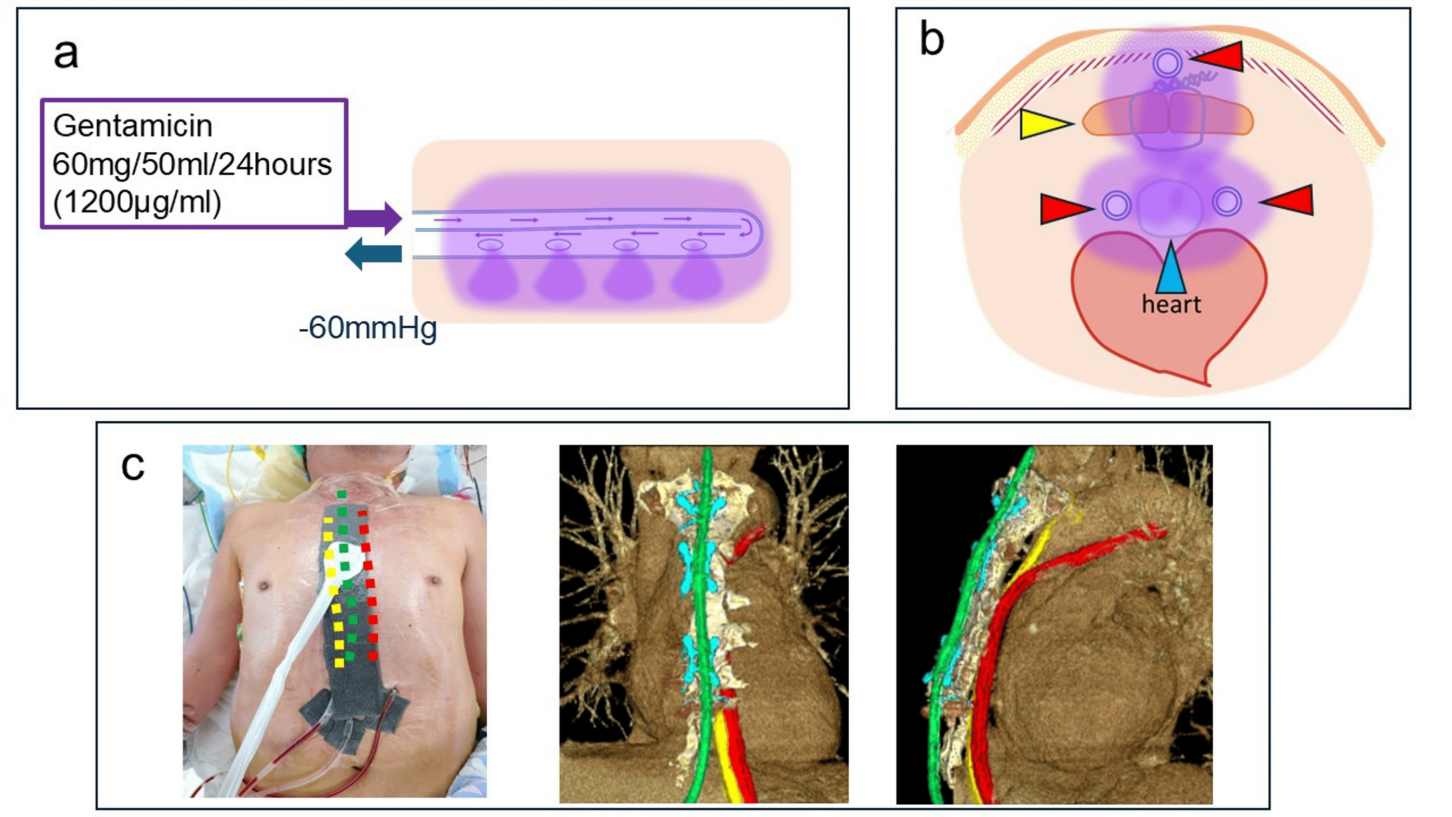
Route of gentamicin administration. (a) Schematic illustration of the CLAP system showing the lumen of the infusion tube. Gentamicin (60 mg/50 mL/24 hours; 1,200 μg/mL) was continuously infused under negative pressure (-60 mmHg). (b) Schematic representation of the tube placement and distribution of the antibiotic. Red arrowheads indicate the CLAP tubes, the yellow arrowhead shows the sternum, and the blue arrowhead indicates the artificial graft. (c) Clinical example of CLAP tube placement. The yellow line represents the space behind the sternum, the red line shows the area around the artificial graft, and the green line indicates the anterior surface of the sternum. CLAP, continuous local antibiotic perfusion

The administration route for antibiotics uses a double-lumen tube called a sump tube (Figure 1), which is designed to prevent excessive negative pressure in the main route by allowing air intake through the air vent route. The antibiotics are injected through the air vent route, and negative pressure is applied to the main route. This method ensures that the antibiotics infiltrate the tissues surrounding the target area through the side holes in the main route, as demonstrated in experimental studies. Gentamicin was prepared by diluting one ampoule (60 mg, 2 mL) with 50 mL of normal saline and was continuously administered over 24 h. Negative pressure of 60 mmHg was applied to the main route for drainage (Figure 1). The following discussion outlines the construction of the CLAP system. Accordingly, the Salem sump tube was placed in the area where gentamicin infiltration was desired. Given that the antibiotic infiltrates only through the sections with side holes, additional side holes were created as needed. Negative pressure was then applied to the main route of the Salem sump tube. When two or more tubes were used, they were connected via Y-connectors and attached to the RENASYS negative pressure wound therapy (NPWT) device (Figure 2).

A drainage bottle was inserted between the RENASYS system and the tubes because the RENASYS canister, known as the “reservoir,” was not precise enough for accurate measurement of drainage volume. The blue air vent route of the Salem sump tube was slightly cut to attach a three-way stopcock, to which the gentamicin infusion route was connected. Each tube was connected to a separate syringe pump for gentamicin administration. One syringe pump was assigned per tube to ensure accurate and steady infusion of the antibiotic. In this study, off-label use refers to the application of the Salem sump tube within the mediastinum as part of the CLAP procedure and the nonstandard connection of the RENASYS canister. Given that the use of the Salem sump tube in such cases is considered off-label, approval from our institutional ethics committee was obtained.

As this was the first application of CLAP for mediastinitis at our institution, there was no prior clinical experience to guide the treatment duration. Therefore, we referred to previous reports of CLAP use in orthopedics, where treatment outcomes have been well established, and adopted a two-week treatment protocol accordingly. Blood cultures were obtained approximately one week after initiating CLAP to assess the treatment response. Wound cultures were not repeated during CLAP because they are not routinely performed once mediastinitis has been confirmed. After completion of CLAP, none of the patients showed fever or elevated inflammatory markers; therefore, repeat blood or wound cultures were not obtained.

Statistical analysis

All data were obtained from the patients’ medical records. Continuous variables were presented as mean±standard deviation (range). The three-year survival rate and rate of freedom from reinfection were analyzed using the Kaplan-Meier method. All statistical analyses were performed using SPSS software version 22.0 (IBM Corp., Armonk, NY, United States). Descriptive statistics were used, given the small sample size and absence of between-group comparisons.

Ethical approval

This study was approved by the institutional ethics committee (Approval No. 2025-19), and written informed consent was obtained from all patients. In addition, because the use of a double-lumen tube (sump tube) was off-label, separate approval for its application was also obtained from the ethics committee (Approval No. 23-13).

## Results

The characteristics of the included patients are detailed in Table 1.

**Table 1:** Patient characteristics. TVR, tricuspid valve replacement; AVR, aortic valve replacement; MVP, mitral valve repair; TAP, tricuspid valve annuloplasty; TARFET, total arch replacement with frozen elephant trunk; CABG, coronary artery bypass grafting; MRSA, methicillin-resistant Staphylococcus aureus

No	Age (years)	Sex	Preoperative diagnosis	Operative procedure	Causative organism	Time to CLAP initiation (days)
1	2	Male	Tetralogy of Fallot	Intracardiac repair	MRSA	10
2	34	Male	Tetralogy of Fallot	Intracardiac repair	MRSA	10
3	40	Male	Transposition of the great arteries	Re-Musturd, TVR	Pseudomonas aeruginosa	17
4	75	Male	Mitral regurgitation	AVR, MVP, TAP	MRSA	22
5	69	Male	Acute type A aortic dissection	TARFET	MRSA	37
6	76	Female	Mitral regurgitation	MVR, TAP	Pseudomonas aeruginosa	29
7	76	Male	Acute myocardial infarction	CABG	MRSA	3

No in-hospital deaths were observed, and all patients achieved wound closure. In the first case, omental filling was performed prior to closure, but this was not required for the other patients. Extubation was achieved an average of 2.2±1.5 days after sternal closure using the CLAP system, and oral intake was resumed at an average of 3.3±1.6 days after closure. The average duration of systemic antibiotic administration was 29.9±14.1 days. The median serum concentration of gentamicin was 1.4 (0.4-2.2) µg/mL. No side effects related to gentamicin administration were observed.

**Figure 3:**
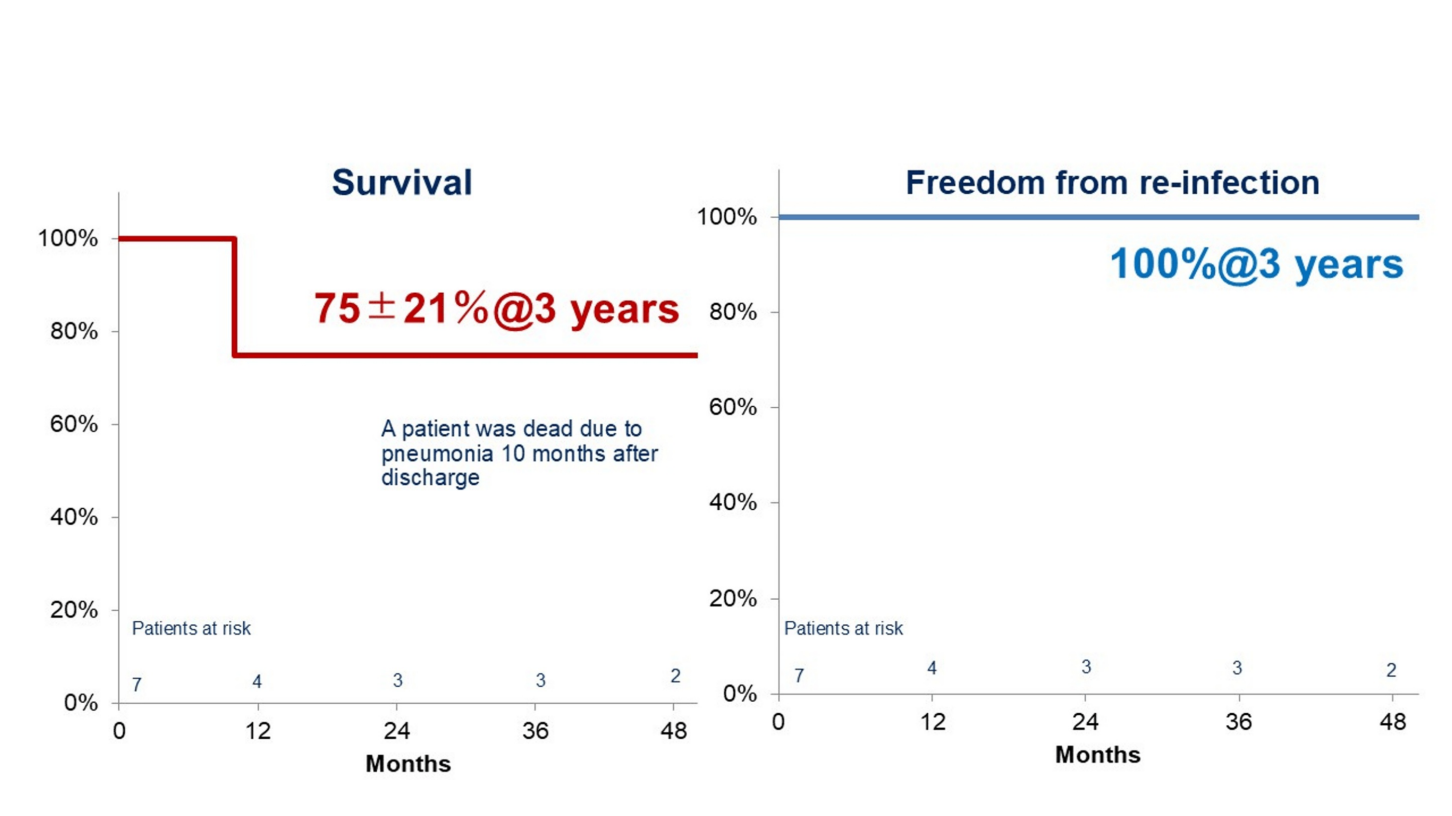
Kaplan-Meier curves showing the three-year overall survival rate and three-year reinfection-free survival rate following CLAP therapy. CLAP, continuous local antibiotic perfusion

None of the patients suffered distant mortality due to reinfection, although one patient passed away from aspiration pneumonia 10 months after surgery. No recurrences of mediastinitis were observed during the study. The Kaplan-Meier survival curve presented in Figure 3 shows a three-year survival rate of 75% and a reinfection-free rate of 100%.

## Discussion

According to the Japan Cardiovascular Surgery Database, the incidence of mediastinitis and sternal osteomyelitis after cardiovascular surgery ranged from 1.5% to 1.9%. However, the same database showed that the in-hospital mortality rate reached 22.9%, with 20% of patients requiring long-term hospitalization of more than 90 days, making it a significant clinical issue [1].

Postoperative mediastinitis was diagnosed following conventional methods, including physical findings, purulent discharge, and imaging studies. As a basic principle, once a diagnosis of mediastinitis had been established, the surgical wound was opened, after which drainage and debridement were performed. The goal of this procedure was to reduce the bacterial load, with achieving sterility not being required, and to preserve the sternum and grafts as much as possible. Drains were then placed to enable the use of CLAP, after which the chest was closed. Thereafter, systemic antibiotics targeting the identified causative organisms were administered. The duration of CLAP use was generally set at two weeks. After this period, the drains were removed, and CLAP was concluded. In our cohort, all patients demonstrated a favorable clinical response to CLAP, consistent with our expectations. Because CLAP was newly introduced at our institution, we established a predefined fallback strategy in the event of insufficient therapeutic response, which consisted of repeated surgical debridement and lavage followed by omental flap coverage. Even in cases caused by MRSA, CLAP was expected to be effective, as previous studies have reported that high-concentration gentamicin can exert antibacterial activity against MRSA. Therefore, MRSA infection was not considered a contraindication or a factor likely to diminish the therapeutic effect of CLAP.

We believe that the fundamental principle for treating postoperative mediastinitis involves surgical wound opening, drainage, and debridement. However, no standardized criteria regarding wound management and closure have been established [6]. Previous studies have demonstrated the effectiveness of continuous suction irrigation for preventing surgical site infections, and other studies have highlighted the efficacy of omental flap coverage. Moreover, studies on continuous irrigation have shown that the average duration from re-thoracotomy to chest closure was 31 days [2]. However, with our method, chest closure was possible immediately after re-thoracotomy. Hence, earlier weaning from mechanical ventilation, resumption of oral intake, and commencement of rehabilitation could be achieved, which we believe contributed to improved infection control.

CLAP was initially developed in orthopedics as a treatment for contaminated wounds, such as open fractures. For bone infections, dedicated pins are inserted into the medullary cavity to allow intramedullary perfusion of gentamicin. Simultaneously, a Salem sump tube is placed in the surrounding soft tissue and connected to negative pressure drainage, which is essential for removing hematomas and debris. Additionally, perfusing gentamicin through the side lumen of the Salem sump enables effective antibiotic infiltration into the surrounding soft tissues. One study reported that among 40 patients with fracture-related infections treated using this method, 35 were able to preserve their implants, whereas 38 achieved fracture healing [4].

In cardiovascular surgery, Shumacker et al. previously reported a method involving the infusion and drainage of saline containing antibiotics into closed surgical wounds [7]. However, the antibiotic concentrations achieved were lower than those achieved through systemic administration, and drug inactivation after solution preparation became a concern. Since then, several studies have evaluated the benefits of irrigation with antibiotic or antiseptic solutions. Other methods for local antibiotic delivery have also been reported, such as the use of Collatamp G® (Innocoll), a type I collagen sponge impregnated with gentamicin, for treating surgical site infections. Randomized controlled trials have shown that prophylactic use of Collatamp G® significantly reduces postoperative infections [8]. This approach has also been used as adjunctive treatment to other procedures, such as pectoralis muscle flap filling, placement of multi-perforated catheters, and NPWT, in some cases with deep sternal wound infections, ultimately allowing successful sternal closure. When Collatamp G® was used at the time of wound closure, the gentamicin concentration in drain fluid remained at 600 and 300 mg/L at 12 and 36 hours after surgery, respectively, which was 75 to 200 times the minimum inhibitory concentration [9]. These concentrations are reportedly bacteriostatic even against organisms with limited susceptibility. In our CLAP method, gentamicin concentrations were continuously maintained at a therapeutic level throughout the procedure, which we believe offers even more effective infection control [10].

Omental filling and musculocutaneous flaps have been reported to be effective in treating severe deep sternal wound infections and prosthetic graft infections [3]. Commonly used musculocutaneous flaps include the pectoralis major, rectus abdominis, and latissimus dorsi muscles. These pedicled flaps can maintain their blood supply and effectively fill large defects, making them useful not only for infection control but also for reconstructing extensive anterior chest wall defects. However, sternal reconstruction using musculocutaneous flaps has been associated with complications such as chronic pain, sternal instability, and long-term muscle weakness [4]. Given the increase in the number of elderly patients undergoing open-heart surgery in recent years, patient frailty has become a major concern. Therefore, preserving function as much as possible and initiating early rehabilitation is desirable when treating deep sternal wound infections and postoperative mediastinitis. CLAP offers a significant advantage in that it enables both functional preservation and early mobilization.

This study has several limitations. First, it was conducted at a single center and included a very small number of patients. Although the CLAP procedure was also applied in patients with prosthetic grafts, determining whether the infection originated from the graft itself or from adjacent mediastinitis was difficult. Thus, no definitive conclusions can be made regarding the effectiveness of CLAP for prosthetic graft infections. Finally, the use of the Salem sump tube in the CLAP system was considered off-label, and the RENASYS device used for NPWT was connected in a manner different from its originally intended use. Nonetheless, this procedure was performed with the approval of the institutional ethics committee and after obtaining informed consent from the patients.

## Conclusions

The outcomes of CLAP for postoperative mediastinitis were favorable, demonstrating that the aim of the study was achieved. CLAP enabled early mobilization, early initiation of oral intake, and a reduced duration of sternal opening, all of which likely contributed to improved infection control and overall clinical recovery. Given the very limited number of cases included herein, further investigations with a larger number of cases are necessary to validate these findings.
